# The Physiology of the Ductless Glands

**Published:** 1901-05-04

**Authors:** 


### THE PHYSIOLOGY OF THE DUCTLESS GLANDS.

So much has been heard of late, and we may add so much has been hoped, regarding the efficacy of organo-therapy, that it is as well to remember how little we know regarding the various "extracts" of the different organs which are now so often employed in the treatment of disease. In an article on "The Physiology of the Ductless Glands," Dr. John Rose Bradford [1] describes the activities of glands as taking three forms: excretion, external secretion, and internal secretion. The fundamental distinction between an excretion and a secretion would seem, he says, to rest in the fact that in the former the products of the excretion are not elaborated by the activity of the gland protoplasm but are simply removed by it from the blood stream. Thus we may describe glands as having one or more than one of these three functions. Some glands confine their activity to the production of an external secretion, as for example the salivary glands; others, such as the thyroid and the supra-renal, produce simply internal secretions so far as is known at present; others possess not only an external but an internal secretion. The most conspicuous instance of this is the pancreas. The same is also true of the liver, since the biliary function is very largely a secretion, and the glycogenic function of the liver is essentially of the nature of an internal secretion. The kidney is a most marked example of a gland having an excretory function.

Many glands whose normal activity is secretory, may under abnormal circumstances also excrete. Thus the salivary glands will excrete iodide of potassium when this is present in the blood stream, and the liver will excrete a large number of drugs and toxic substances which are being absorbed from the alimentary canal.

In many glands the phenomena of secretion are under the control of the nervous system, as, for example, in the case of salivary and sweat glands. In the process of external secretion there is a gradual elaboration or storing up in the secreting cell of an antecedent of the specific constituent of the secretion. This is a slow process which is carried on especially during the periods of rest. But at the moment of secretion there is a sudden conversion of this antecedent into the actual constituent, and at the same time a considerable elimination of water, and these latter processes are especially under the control of the nervous system.

This nervous influence is accompanied by changes in the blood-vessels, but in the case of external secretion there is good evidence that these two processes, the vascular and the nervous, although occurring together, are essentially independent. In the case of the internal secretions the part played by the nervous system is more obscure, but in certain glands, as, for instance, the liver and even the thyroid, there is evidence that excitation or destruction of nerves and nerve centres is capable of producing profound effects on the activity of the internal secretion. When we come to consider what are these internal secretions, we find Dr. Bradford saying that they seem to be substances required for the physiological activity of certain specific tissues of the body; thus the glycogen and sugar elaborated by the liver are especially required for the metabolism of muscle, the thyroidin elaborated by the thyroid may be especially necessary for the maintenance of the normal activities of the central nervous system, and the material formed by the supra-renal would seem to be required, or at any rate to be very necessary, for the maintenance of the activity of the involuntary muscular tissue of the blood-vessels.

Thus we see a strict analogy between internal and external secretion, the substance produced being in each case manufactured by the gland cells, and required for the performance of function by other organs; in the one case, however, being transported to these distant parts by the blood-stream, in the other being carried there by special ducts provided for the purpose.

There is this contrast, however, to be drawn between them, namely, that an external secretion contains in most cases a member of the group of ferments, and only in a few instances contains a definite complex substance, whereas in the case of internal secretions it is more usual for the substance to be of the latter nature, and it is only in exceptional cases that it is of the nature of a ferment. In regard to the rôle played by these substances which form the essential parts of external secretions, evidence chiefly centres round the thyroid gland, in regard to which there is much, both clinical and experimental, to show that diseased condition may be produced either by the arrest of the secretion or by its formation in excessive amount, or, perhaps, owing to its containing abnormal constituents. Cretinism, myxœdema, and exophthalmia goitre afford illustrations of these points.

When we turn from the thyroid to other glands the rôle played by these in the production of disease is much more obscure. It has been supposed that some of the phenomena of Addison's disease can be accounted for by the suppression of the internal secretion of these glands, and no doubt the circulatory weakness and general muscular debility seen in this malady may be thus accounted for, and there are other points which seem to confirm this hypothesis. On the other hand no physiologist has been able to produce any condition closely analogous to Addison's disease by lesions of the supra-renal, nor is the use of supra-renal extract of much service in the treatment of the malady, and it seems impossible at the present time to give a complete explanation of the pathology of Addison's disease based on the known functions of the supra-renal. In regard to other glands both the physiology and the pathology of internal secretions become less definite, and there is room for great difference of opinion in the interpretation of the phenomena, as is seen every day in the discussions concerning the processes engaged in the production of glycosuria.

So far as morbid anatomy is concerned there would seem a close relation between the pituitary body and certain diseases of the skeletal structures, but at the present moment there is no physiological evidence to show in what way diseases of the pituitary body give rise to these disorders of nutrition, and it is to be noted that the pituitary body has been completely removed without producing any such changes.

As to the kidney, it is clear from Dr. Rose Bradford's own experiments that the renal tissues must have some other functions than those of mere excretion, but they do not afford conclusive evidence of an internal secretion, and treatment with renal extracts has not hitherto been followed by any great success.

Again, there is much to support the presumption of an internal secretion by the testes and ovaries in the results which are known to follow removal of these glands; but then, while it is possible that such efforts as these may be dependent on internal secretions, it is also possible that they may be produced in some remote way through the intermediation of the nervous system. A great deal has been done lately, and is now being done, in the treatment of disease by the administration of organic products, but when we find people arguing by analogy from thyroid to supra-renals and other glands, and then to extracts of organs which are not glands in the ordinary meaning of the term, it is well to turn to Dr. Bradford's calm review of the whole subject from the stand-point of the physiologist, from which we see that however much we may hope, and however astutely we may guess, our *knowledge* on the subject of organo-therapy does not at the present go very deep.

[1] Practitioner, April.

